# Supplementary material for: Unveiling the Bioactive Potential of the Invasive Jellyfish Phyllorhiza punctata Through Integrative Transcriptomic and Proteomic Analyses
Source: Biomolecules. 2025 Aug 4;15(8):1121. doi: 10.3390/biom15081121 (PMC12383608; doi:10.3390/biom15081121)
Supplement: Supplementary file 1 [file biomolecules-15-01121-s001.zip › Suplementary Material/FileS3/PEPFOLD4/XP_065061803_7839/clusters.html]

 PEP-FOLD clusters


Download archive of all models  here   

|  |  |  |
| --- | --- | --- |
| # | file | sOPEP |
| # cluster 1 - Size: 1 |  |  |
|  | XP\_065061803\_7839-model1 | -34.7306 |
| # cluster 2 - Size: 11 |  |  |
|  | XP\_065061803\_7839-model2 | -31.5146 |
|  | XP\_065061803\_7839-model2.1 | -30.9911 |
|  | XP\_065061803\_7839-model2.2 | -30.9726 |
|  | XP\_065061803\_7839-model2.3 | -30.9005 |
|  | XP\_065061803\_7839-model2.4 | -30.1961 |
|  | XP\_065061803\_7839-model2.5 | -29.8558 |
|  | XP\_065061803\_7839-model2.6 | -29.7299 |
|  | XP\_065061803\_7839-model2.7 | -29.5986 |
|  | XP\_065061803\_7839-model2.8 | -29.5827 |
|  | XP\_065061803\_7839-model2.9 | -29.1781 |
|  | XP\_065061803\_7839-model2.10 | -28.5798 |
| # cluster 3 - Size: 36 |  |  |
|  | XP\_065061803\_7839-model3 | -31.5021 |
|  | XP\_065061803\_7839-model3.1 | -30.9201 |
|  | XP\_065061803\_7839-model3.2 | -30.6748 |
|  | XP\_065061803\_7839-model3.3 | -30.6229 |
|  | XP\_065061803\_7839-model3.4 | -30.5079 |
|  | XP\_065061803\_7839-model3.5 | -30.4896 |
|  | XP\_065061803\_7839-model3.6 | -30.4783 |
|  | XP\_065061803\_7839-model3.7 | -30.4152 |
|  | XP\_065061803\_7839-model3.8 | -30.3692 |
|  | XP\_065061803\_7839-model3.9 | -30.367 |
|  | XP\_065061803\_7839-model3.10 | -30.3167 |
|  | XP\_065061803\_7839-model3.11 | -30.2934 |
|  | XP\_065061803\_7839-model3.12 | -30.2871 |
|  | XP\_065061803\_7839-model3.13 | -30.2734 |
|  | XP\_065061803\_7839-model3.14 | -30.2539 |
|  | XP\_065061803\_7839-model3.15 | -30.1936 |
|  | XP\_065061803\_7839-model3.16 | -30.1575 |
|  | XP\_065061803\_7839-model3.17 | -30.0651 |
|  | XP\_065061803\_7839-model3.18 | -30.0381 |
|  | XP\_065061803\_7839-model3.19 | -30.0165 |
|  | XP\_065061803\_7839-model3.20 | -29.9966 |
|  | XP\_065061803\_7839-model3.21 | -29.9791 |
|  | XP\_065061803\_7839-model3.22 | -29.9738 |
|  | XP\_065061803\_7839-model3.23 | -29.9648 |
|  | XP\_065061803\_7839-model3.24 | -29.9508 |
|  | XP\_065061803\_7839-model3.25 | -29.8462 |
|  | XP\_065061803\_7839-model3.26 | -29.7564 |
|  | XP\_065061803\_7839-model3.27 | -29.6911 |
|  | XP\_065061803\_7839-model3.28 | -29.5394 |
|  | XP\_065061803\_7839-model3.29 | -29.5346 |
|  | XP\_065061803\_7839-model3.30 | -29.5101 |
|  | XP\_065061803\_7839-model3.31 | -29.464 |
|  | XP\_065061803\_7839-model3.32 | -29.3722 |
|  | XP\_065061803\_7839-model3.33 | -29.3102 |
|  | XP\_065061803\_7839-model3.34 | -29.1259 |
|  | XP\_065061803\_7839-model3.35 | -28.8558 |
| # cluster 4 - Size: 3 |  |  |
|  | XP\_065061803\_7839-model4 | -31.3528 |
|  | XP\_065061803\_7839-model4.1 | -29.6842 |
|  | XP\_065061803\_7839-model4.2 | -29.5293 |
| # cluster 5 - Size: 6 |  |  |
|  | XP\_065061803\_7839-model5 | -31.3055 |
|  | XP\_065061803\_7839-model5.1 | -30.6357 |
|  | XP\_065061803\_7839-model5.2 | -30.6109 |
|  | XP\_065061803\_7839-model5.3 | -30.1293 |
|  | XP\_065061803\_7839-model5.4 | -29.6036 |
|  | XP\_065061803\_7839-model5.5 | -29.5861 |
| # cluster 6 - Size: 5 |  |  |
|  | XP\_065061803\_7839-model6 | -30.9899 |
|  | XP\_065061803\_7839-model6.1 | -30.2247 |
|  | XP\_065061803\_7839-model6.2 | -29.4657 |
|  | XP\_065061803\_7839-model6.3 | -29.323 |
|  | XP\_065061803\_7839-model6.4 | -29.19 |
| # cluster 7 - Size: 11 |  |  |
|  | XP\_065061803\_7839-model7 | -30.9686 |
|  | XP\_065061803\_7839-model7.1 | -30.8288 |
|  | XP\_065061803\_7839-model7.2 | -30.3853 |
|  | XP\_065061803\_7839-model7.3 | -30.2617 |
|  | XP\_065061803\_7839-model7.4 | -30.0907 |
|  | XP\_065061803\_7839-model7.5 | -29.9451 |
|  | XP\_065061803\_7839-model7.6 | -29.534 |
|  | XP\_065061803\_7839-model7.7 | -29.4877 |
|  | XP\_065061803\_7839-model7.8 | -29.3038 |
|  | XP\_065061803\_7839-model7.9 | -29.1634 |
|  | XP\_065061803\_7839-model7.10 | -28.7831 |
| # cluster 8 - Size: 2 |  |  |
|  | XP\_065061803\_7839-model8 | -30.6766 |
|  | XP\_065061803\_7839-model8.1 | -29.2149 |
| # cluster 9 - Size: 4 |  |  |
|  | XP\_065061803\_7839-model9 | -30.3709 |
|  | XP\_065061803\_7839-model9.1 | -30.3141 |
|  | XP\_065061803\_7839-model9.2 | -29.8144 |
|  | XP\_065061803\_7839-model9.3 | -29.2613 |
| # cluster 10 - Size: 2 |  |  |
|  | XP\_065061803\_7839-model10 | -30.2242 |
|  | XP\_065061803\_7839-model10.1 | -29.572 |
| # cluster 11 - Size: 1 |  |  |
|  | XP\_065061803\_7839-model11 | -30.1508 |
| # cluster 12 - Size: 3 |  |  |
|  | XP\_065061803\_7839-model12 | -30.0617 |
|  | XP\_065061803\_7839-model12.1 | -29.6412 |
|  | XP\_065061803\_7839-model12.2 | -29.5989 |
| # cluster 13 - Size: 1 |  |  |
|  | XP\_065061803\_7839-model13 | -29.9966 |
| # cluster 14 - Size: 2 |  |  |
|  | XP\_065061803\_7839-model14 | -29.9057 |
|  | XP\_065061803\_7839-model14.1 | -29.8754 |
| # cluster 15 - Size: 2 |  |  |
|  | XP\_065061803\_7839-model15 | -29.8915 |
|  | XP\_065061803\_7839-model15.1 | -29.4762 |
| # cluster 16 - Size: 1 |  |  |
|  | XP\_065061803\_7839-model16 | -29.8865 |
| # cluster 17 - Size: 1 |  |  |
|  | XP\_065061803\_7839-model17 | -29.8339 |
| # cluster 18 - Size: 1 |  |  |
|  | XP\_065061803\_7839-model18 | -29.7184 |
| # cluster 19 - Size: 2 |  |  |
|  | XP\_065061803\_7839-model19 | -29.5412 |
|  | XP\_065061803\_7839-model19.1 | -29.307 |
| # cluster 20 - Size: 2 |  |  |
|  | XP\_065061803\_7839-model20 | -29.5263 |
|  | XP\_065061803\_7839-model20.1 | -29.1334 |
| # cluster 21 - Size: 2 |  |  |
|  | XP\_065061803\_7839-model21 | -29.3833 |
|  | XP\_065061803\_7839-model21.1 | -28.9862 |
| # cluster 22 - Size: 1 |  |  |
|  | XP\_065061803\_7839-model22 | -28.7118 |
|  |  |  |
